# Review of fibromyalgia treatment guidelines: part 2 – pharmacological treatment

**DOI:** 10.1186/s42358-025-00483-2

**Published:** 2026-01-22

**Authors:** Roberto Ezequiel Heymann, Marcelo Cruz Rezende, Eduardo dos Santos Paiva, Marcos Renato de Assis, Alessandra de Sousa Braz, Aline Ranzolin, Ana Paula Monteiro Gomides Reis, Andrea Pimentel Fonseca Golmia, Anna Beatriz Assad Maia, Denison Santos Silva, Fernando Augusto Chiuchetta, Gabriela Galassi, Izabela Guimarães, José Eduardo Martinez, José Roberto Provenza, Juliana Maria de Freitas Trindade Costa, Marcos Paulo Veloso Correia, Marco Antônio Gonçalves Pontes Filho, Marco Aurelio Goldenfum, Marcos Aurelio de Freitas Machado, Melissa Mariti Fraga, Nilton Salles Rosa Neto, Rafael Navarrete Fernandez

**Affiliations:** 1 Hospital Israelita Albert Einstein, Sâo Paulo, Brazil; 2 Pain specialist certified by The Brazilian Medical Association, Sâo Paulo, Brazil; 3 Universidade Federal do Paranâ, Curitiba, Brazil; 4 Faculdade de Medicina de Assis, Sâo Paulo, Brazil; 5 Hospital Universitârio Lauro Wanderley, Universidade Federal da Paraíba, Joâo Pessoa, Brazil; 6 Hospital das Clínicas de Pernambuco, Recife PE, Brazil; 7 Centro Universitário de Brasília, Brasília, Brazil; 8 Hospital Israelita Albert Einstein, São Paulo, Brazil; 9 University Hospital of Brasília, Brasília, Brazil; 10 Universidade Tiradentes, Aracaju, Sergipe Brazil; 11 Hospital da XV, Curitiba, Brazil; 12 Biomedical Researcher Axia Bio, Sâo Paulo, Brazil; 13 Pain specialist certified by the Brazilian Medical Association (AMB), Sâo Paulo, Brazil; 14 Pontifícia Universidade Católica de São Paulo, Sâo Paulo, Brazil; 15 Pontifícia Universidade Católica de Campinas, Sâo Paulo, Brazil; 16 Hospital de Aeronâutica de Recife, Recife, Brazil; 17 Universidade Federal do Rio de Janeiro, Rio de Janeiro, Brazil; 18 Pain specialist certified by the Brazilian Medical Association (AMB), Sâo Paulo, Brazil; 19 Pontifícia Universidade Católica do Rio Grande do Sul, Porto Alegre, Rio Grande do Sul Brazil; 20 Member of the International Association for the Study of Pain (IASP), Sâo Paulo, Brazil; 21 Universidade Federal de Sâo Paulo, Sâo Paulo, Brazil; 22 Universidade Santo Amaro, Sâo Paulo, Brazil; 23 Pontificia Universidade Católica de Goiâs, Goiânia, Brazil

**Keywords:** Fibromyalgia, Chronic pain, Central sensitization, Guidelines, Pharmacological treatment, Duloxetine, Tricyclic antidepressants (TCA), Serotonin-norepinephrine reuptake inhibitors (SNRI), Pregabalin, Gabapentin

## Abstract

Fibromyalgia is a complex condition characterized by widespread chronic pain, fatigue, and sleep disturbances, which significantly affect quality of life. Its pathophysiology involves central sensitization (CS) and changes in neurotransmitters. Management is challenging and requires a multidisciplinary approach that integrates pharmacological and nonpharmacological treatments. This consensus, which is based on the BASCE system, presents updated guidelines for drug and nondrug treatments. The methodology focused on the evaluation of meta-analyses and systematic reviews of the literature and on voting by experts from the Brazilian Society of Rheumatology, ensuring recommendations with at least 70% agreement. Divided into two articles, this article addresses pharmacological strategies, incorporating recent advances to optimize clinical management and promote uniformity in professional practice.

## Introduction

Fibromyalgia (FM) is a complex clinical syndrome characterized by chronic widespread pain, fatigue, sleep disturbances, and other associated symptoms that significantly impair patients’ quality of life. Although the underlying pathophysiological mechanisms are not yet fully elucidated, current evidence suggests that central sensitization (CS) and dysregulation of neurotransmitter systems play a crucial role in the initiation and maintenance of FM symptoms [1, 2].

The management of FM remains challenging due to its multifactorial etiology and the heterogeneous response to available therapeutic modalities. Pharmacological treatment primarily aims to alleviate symptoms, thereby improving functionality and quality of life. However, pharmacotherapy alone is insufficient; an integrated, multimodal approach—including physical activity, patient education, and psychosocial interventions—is essential for optimal disease management.

This article, as part of the revised guidelines for FM treatment, aims to update the 2010 recommendations [3] by integrating the latest scientific evidence to support the rational use of pharmacological agents in FM management. The proposed recommendations are designed to enhance clinical decision-making, promote safe and effective therapeutic strategies, and contribute to greater standardization in professional practice for improved patient care.

For clarity and better organization, the consensus on FM treatment has been structured into two complementary articles. The first article focuses on patient monitoring strategies, highlighting the importance of individualized and continuous follow-up, as well as evidence-based nonpharmacological interventions. This second article provides a comprehensive update on pharmacological management, integrating the most recent advances in the field.

## Materials and methods

The methodology employed in this consensus was based on the BASCE System (an acronym for Search, Analyze, Select, Classify, and Elaborate) [4], an organizational framework developed by the consulting firm Axia.Bio. This system aims to minimize biases and deviations in the results by applying criteria derived from well-established scientific literature. It provides a systematic approach to consensus-building, ensuring the relevance of recommendations to the local context. In this study, the methodology prioritized the evaluation of systematic reviews and meta-analyses, supplemented by expert voting within the Brazilian Society of Rheumatology (SBR) to formulate evidence-based recommendations.

## Methodological steps

The consensus process was structured into the following key steps:

1. Comprehensive and systematic literature search – A search for meta-analyses and systematic reviews related to fibromyalgia (FM) was conducted.
2. Structured evaluation of studies – A panel of local experts (Group I) reviewed and selected the most relevant literature based on predefined questions, assigning scores for prioritization.
3. Expert review and voting – A second expert group (Group II) analyzed the recommendations derived from the selected studies, considering their applicability in Brazil. Voting was conducted to ensure consensus.

The process was divided into two major phases:

First Phase: Formulation of Questions and Literature Review

A total of 31 key questions were formulated by SBR experts of group I, focusing on therapeutic monitoring and treatment strategies for FM. Based on these questions, a systematic bibliographic search was performed in databases such as PubMed, LILACS, and the Cochrane Library, employing specific search strategies.

The PubMed and LILACS search followed the strategy:

(“fibromyalgia” [MeSH Terms] OR “fibromyalgia”[All Fields]) AND systematic[sb] AND ((“1”[PDAT]: “2008/06/13”[PDAT]) AND (English[lang] OR Spanish[lang] OR Portuguese[lang]))

This strategy yielded 238 publications. In the Cochrane Library, the search term *fibromyalgia* resulted in 51 publications.

After eliminating duplicate references, the relevant studies were forwarded to a panel of six experts (Group I), who assessed and voted on the relevance of each study to address the formulated questions. Only studies that achieved at least 70% agreement among the experts proceeded to the next stage. Additionally, experts considered the level of recommendation and applicability of the findings in the Brazilian context when selecting references.

Second Phase: Expert Voting on Recommendations During the second phase, a broader panel of FM experts convened to vote on whether to accept or reject the proposed recommendations based on the selected studies. Group II comprised members of Group I along with additional 16 FM experts, totaling 22 specialists, all affiliated with the Brazilian Society of Rheumatology.

1. Voting was conducted electronically and anonymously using the Mentimeter platform (https://www.mentimeter.com).
2. Statements achieving at least 70% agreement (YES or NO) were accepted as recommendations.
3. Statements failing to reach consensus in the first round were subjected to expert debate, followed by a second voting round.
4. Statements not reaching the required 70% threshold in the second round were excluded from the final recommendations.

All 31 proposed questions reached expert consensus, ensuring that the endorsed recommendations were supported by at least 70% agreement among participants and were backed by bibliographic references. It is important to emphasize that the absence of a recommendation for a particular treatment does not imply its inefficacy or contraindication. Rather, this consensus exclusively endorses treatments supported by robust scientific evidence.

All voting records and results were documented and archived via the Mentimeter platform, ensuring transparency and traceability.

This article specifically addresses pharmacological treatment strategies for FM, corresponding to the last 13 questions of the 31 evaluated in the consensus process.

## Recommendations

Are amitriptyline and other tricyclic antidepressant drugs effective in the treatment of FM?

Consensus: There is evidence to recommend Amitriptyline for the treatment of FM. No evidence was found for other tricyclic antidepressants.

Amitriptyline is recognized as an effective pharmacological option for the treatment of fibromyalgia (FM) and is recommended in clinical guidelines, particularly at doses ranging from 25 a 50 mg/day. Although the available evidence is limited, studies indicate that amitriptyline can significantly improve key symptoms of FM, including pain, sleep disturbances, fatigue, and overall quality of life [5–7].

Amitriptyline exerts its effects by modulating the neurotransmitters serotonin and norepinephrine, which are crucial pathways involved in pain modulation and the regulation of sleep. These actions contribute to pain relief and improved sleep quality, which are essential components in the management of FM symptoms. Studies have reported that 30% to 50% of FM patients respond positively to amitriptyline, underscoring its utility as a therapeutic alternative, particularly for those who do not achieve satisfactory outcomes with other treatments [8]. However, its efficacy in addressing fatigue and associated cognitive deficits remains uncertain and warrants further investigation through additional long-term studies [9].

Meta-analyses and systematic reviews have demonstrated that tricyclic antidepressants, including amitriptyline, produce a moderate effect on pain reduction and improvement of sleep quality, although they exert a less pronounced impact on fatigue and patients’ overall quality of life [5–7]. A specific meta-analysis highlighted that, compared to placebo, amitriptyline provides significant benefits in terms of pain relief and sleep improvement. However, tolerability can be a concern due to adverse effects, such as dry mouth, constipation, and weight gain, which may limit its use in certain patients [5].

Are muscle relaxants recommended for the treatment of FM?

Consensus: There is evidence to recommend the use of cyclobenzaprine for the treatment of FM. No evidence was found for other muscle relaxants

Cyclobenzaprine is considered a secondary pharmacological option for the treatment of fibromyalgia (FM), with low doses (5–10 mg at bedtime) demonstrating moderate efficacy in improving sleep quality, particularly in patients with comorbid sleep disorders. However, its effects on pain and fatigue remain inconsistent across studies [10, 11].

Recent investigations have explored the potential of a sublingual formulation of cyclobenzaprine (TNX-102 SL), which has demonstrated a significant reduction in daily pain compared with placebo, in addition to improving sleep parameters. Despite these promising findings, adverse effects such as oral hypoesthesia and paresthesia have been reported, underscoring the need for a careful risk-benefit assessment before clinical use [10].

Another pharmacological approach evaluated in FM treatment is the combination of carisoprodol, acetaminophen (paracetamol), and caffeine. This combination has been associated with improvements in pain relief, sleep quality, overall well-being, and increased pain thresholds. However, it is important to note that a significant placebo effect was also observed, particularly in pain reduction and sleep improvement, while no notable effect was reported on general malaise [12].

Given its effects on sleep regulation, cyclobenzaprine may be considered a therapeutic alternative for FM patients with marked sleep disturbances, provided that potential adverse effects are carefully monitored.

Are selective serotonin inhibitor antidepressants (SSRIs) effective in treating FM?

Consensus: There is not enough evidence to recommend SSRI antidepressants for the treatment of FM.

Compared to placebo, selective serotonin reuptake inhibitors (SSRIs) demonstrate a modest effect on pain reduction in patients with fibromyalgia (FM), with a number needed to treat (NNT) of 6.3 (95% CI: 4.1–14.1) for a 30% reduction in pain. However, SSRIs do not show significant benefits in alleviating fatigue or sleep disturbances [5, 13, 14]. Additionally, SSRIs are more effective than placebo in the treatment of depressive symptoms in FM patients, without an associated increase in serious adverse events [14].

Although SSRIs are frequently prescribed for the management of depression in FM, robust evidence supporting their superiority over placebo for pain, fatigue, or sleep disturbances remains lacking [14]. Among the SSRIs, fluoxetine has been shown to provide modest improvements in both pain and depression, though its efficacy does not significantly differ from that of other SSRIs [14].

In clinical practice, the amelioration of depressive symptoms induced by SSRIs may indirectly contribute to overall symptom relief in FM, potentially improving patients’ perceived well-being and quality of life.

Are duloxetine and other dual serotonin-norepinephrine reuptake inhibitors effective in the treatment of FM?

Consensus: There is evidence to recommend duloxetine and milnacipran in the treatment of FM. There is no evidence to recommend venlafaxine or desvenlafaxine for the treatment of FM.

Duloxetine has demonstrated consistent efficacy in relieving fibromyalgia (FM) symptoms compared to placebo, with doses ranging from 30 to 120 mg/day. Among these, the 60 mg/day dose offers the optimal balance between efficacy and safety, although individualized dose adjustments may be necessary. Higher doses (120 mg/day) are associated with an increased incidence of adverse effects, including nausea, dry mouth, insomnia, and dizziness, which contribute to a higher treatment discontinuation rate [15].

Duloxetine is considered moderately effective for FM, providing some improvement in core symptoms. However, the overall quality of evidence remains low, and only a subset of patients derive significant clinical benefit. The number needed to treat (NNT) for ≥ 50% pain relief was 11 (95% CI: 9–14), while for overall symptom improvement, the NNT was 5 (95% CI: 4–8). No clinically relevant effects were observed in relation to fatigue, sleep disturbances, or quality of life. Treatment discontinuation due to adverse effects was reported in approximately 19% of patients receiving duloxetine or milnacipran, with a number needed to harm (NNH) of 14 (95% CI: 10–25). However, no significant increase in serious adverse events was observed compared to placebo [16].

Milnacipran has also been associated with moderate pain relief in FM (NNT 11; 95% CI: 9–14), at effective doses ranging from 100 to 200 mg/day, but it is currently unavailable in Brazil [16].

Regarding venlafaxine and desvenlafaxine, the evidence remains limited. A small study on desvenlafaxine found no significant efficacy compared to placebo but reported good tolerability, with a lower risk of somnolence and insomnia relative to milnacipran [16]. Given the limited efficacy and potential adverse effects, the use of venlafaxine and desvenlafaxine in FM should be approached with caution [16].

Are antidepressants from other classes effective in the treatment of FM? (especially trazodone, bupropion, and mirtazapine).

Consensus: There is no evidence to recommend antidepressants from other classes for the treatment of FM.

Mirtazapine enhances norepinephrine and serotonin release via α2-adrenergic antagonism and blocks 5-HT2/5-HT3 receptors, promoting sedation and sleep, with minimal anticholinergic effects [17]. A systematic review in fibromyalgia (FM) identified three randomized controlled trials and one open-label study (15–30 mg/day), showing improvements in pain [18–21], sleep [19, 20], and fatigue [18], though limited by small samples, short follow-up, and heterogeneous outcome measures. Larger, long-term studies are needed to establish its role [17]. Another review found no significant benefit over placebo in pain, global impression, quality of life, fatigue, or mood, with low to very low evidence quality and potential bias, concluding that risks may outweigh benefits, except in a minority of patients who may respond favorably [22].

Trazodone, though not approved for FM, may help alleviate sleep disturbances and associated symptoms. However, current evidence is limited to open-label studies, and further randomized controlled trials (RCTs) are needed to establish its efficacy and safety in FM [23, 24].

Bupropion, despite its pharmacological properties that could theoretically improve FM symptoms, lacks sufficient clinical evidence to support its routine use in FM treatment.

Are pregabalin and gabapentin effective in the treatment of FM?

Consensus: There is evidence to recommend pregabalin for the treatment of FM, but not enough evidence to recommend gabapentin.

Pregabalin (PGB) is a second-generation antiepileptic that exerts its effects by binding to the α2-δ subunits of voltage-gated calcium channels, thereby reducing calcium influx into nerve terminals. This mechanism modulates neurotransmitter release, including glutamate, norepinephrine, and substance P, which contributes to its analgesic, anticonvulsant, and anxiolytic properties [25, 26].

Evidence suggests that PGB provides greater symptom relief than placebo for up to six months, improving pain, sleep disturbances, quality of life, and fatigue, though its effects on depressed mood remain inconclusive. The number needed to treat (NNT) for a 30% reduction in pain was 8.6 (95% CI: 6.4–12.9), compared with 7.2 (95% CI: 5.2–11.4) for duloxetine (DLX) and 19 (95% CI: 7.4–20.5) for milnacipran (MLN). In terms of treatment discontinuation due to adverse effects, the number needed to harm (NNH) was 14.9 (95% CI: 9.1–41.4) for DLX, 7.6 (95% CI: 6.2–9.9) for MLN, and 7.6 (95% CI: 6.3–9.4) for PGB [27].

A Cochrane review assessing PGB for pain relief in adults with FM included randomized, double-blind trials lasting at least eight weeks, comparing PGB with placebo or other treatments. The analysis found that 39% to 43% of patients on PGB (300–600 mg/day) experienced moderate pain relief, compared to 28% in the placebo group (high-certainty evidence). Similar results were reported for global patient-reported improvement (PGIC: “very much improved”), with NNTs ranging from 7 to 14. Compared to placebo, PGB significantly reduced pain at 12–26 weeks, with adverse events occurring in approximately 10% more participants [28].

Regarding gabapentin, the available evidence remains insufficient to confirm or refute its efficacy at doses of 1200 to 2400 mg/day in FM pain management. A single 12-week trial suggested that a subset of patients may experience pain relief at a maximum dose of 2400 mg/day, but the findings were limited [29].

Despite the scarcity of robust studies, a panel of experts noted that gabapentin has been widely used in clinical practice for FM treatment. Although scientific evidence remains limited, positive outcomes in symptom management have been observed, reinforcing the role of gabapentin as a potential therapeutic option in routine FM care.

Are naltrexone and tramadol effective in the treatment of FM? Can other opioids be used to treat FM?

Consensus: There is not enough evidence to recommend the use of low dose naltrexone or tramadol in the treatment of FM. The use of opioids in FM is not recommended.

Low-dose naltrexone (LDN) is hypothesized to exert its effects in (FM) by inhibiting microglial activation in the central nervous system, thereby reducing central and peripheral inflammation. This mechanism may contribute to symptom relief in FM. A systematic review evaluating nine studies—including one randomized controlled trial (RCT), case reports, case series, and pilot trials—suggested promising results for LDN, with doses ranging from 0.1 mg/day to 9 mg/day, the most common being 4.5 mg/day [30, 31].

One study within the review reported that LDN led to a symptom reduction of more than 30% compared to placebo, with improvements in mechanical pain and heat pain thresholds [31]. Another review similarly suggested potential benefits in pain reduction and quality of life improvement but emphasized key limitations, such as small sample sizes and elevated risk of bias. Despite these preliminary findings, the current evidence remains weak, and larger, well-designed clinical trials are needed to confirm LDNs role in FM management [32].

Tramadol, a weak opioid agonist, has demonstrated modest benefits in FM treatment, primarily due to its dual mechanism of action—serotonin and norepinephrine reuptake inhibition in addition to μ-opioid receptor activity. A systematic review of four trials involving 459 patients found that tramadol combined with a non-opioid analgesic improved quality of life compared to placebo. However, tramadol alone did not demonstrate significant efficacy in FM symptom relief. The GRADE approach rated the quality of this evidence as low, and tramadol was associated with higher treatment discontinuation rates due to adverse effects, though no serious events were reported [33, 34].

Regarding other opioids, no evidence supports their efficacy in FM. Their use has been linked to worse clinical outcomes, including greater pain interference, poorer functional status, and increased symptoms of depression and insomnia. Due to these risks, clinical guidelines strongly advise against opioid use in FM management [35–38].

Are simple analgesics and nonsteroidal anti-inflammatory drugs (NSAIDs) effective in the treatment of FM?

Consensus: There is no evidence to recommend the use of simple analgesics and NSAIDs in the treatment of FM.

The use of NSAIDs in fibromyalgia (FM) has been evaluated in a modest number of studies, which are generally small, methodologically inadequate, and at risk of bias. These findings suggest that NSAIDs are not effective in the treatment of FM [39, 40].

A Cochrane review specifically analyzed NSAID use in FM patients, assessing six randomized studies of low methodological quality, with a total of 292 participants randomized to NSAID or placebo groups. None of the studies evaluated FM-specific outcomes, such as the Fibromyalgia Impact Questionnaire-Revised (FIQR), and instead focused on pain reduction by 30% or 50%. Various NSAIDs were evaluated, including etoricoxib (90 mg/day), ibuprofen (2400 mg/day), naproxen (1000 mg/day), and tenoxicam (20 mg/day). No significant differences were observed between NSAIDs and placebo for any outcome studied [40].

The lack of efficacy of NSAIDs in FM is not unexpected, as FM is primarily considered a non-inflammatory condition. Consequently, most treatment guidelines do not recommend their use. However, it is important to recognize that FM patients frequently experience overlapping regional pain syndromes, such as bursitis, tendinitis, and myofascial pain, which may result from increased pain sensitivity. These conditions are often asymptomatic in individuals without FM but can contribute to additional pain burden in FM patients. In such cases, NSAIDs may be used for short-term pain management.

The use of common analgesics, such as dipyrone and acetaminophen (paracetamol)—often available over the counter and combined with muscle relaxants—is frequent in FM. While there is no robust evidence supporting their efficacy, these agents are safer for long-term use compared to NSAIDs. If patients report symptom relief, particularly when analgesia facilitates greater physical activity engagement and improves daily function, there is no compelling reason to discontinue their use.

Are there pharmacological combinations that have been shown to be effective in FM?

Consensus: Current evidence remains insufficient to support the routine use of combination pharmacotherapy in the treatment of fibromyalgia (FM).

There is a scarcity of large, high-quality studies directly comparing combination pharmacotherapy with monotherapy for the treatment of fibromyalgia (FM). This limitation restricts the scientific basis to support or refute the routine use of combination therapy in FM management [41].

Despite the lack of robust evidence, expert consensus suggests that therapeutic combinations are commonly utilized in clinical practice. When carefully monitored and tailored to individual patient needs, certain combinations may provide symptom relief, particularly in patients with partial responses to monotherapy. However, due to the potential for increased adverse effects and drug interactions, combination therapy should be implemented with caution and adjusted based on efficacy and tolerability.

Do cannabinoids play a role in FM treatment?

Consensus: There is no evidence to recommend the use of cannabinoids in the treatment of FM.

Cannabinoids exert their effects by stimulating CB1 and CB2 receptors of the endocannabinoid system, contributing to antiemetic, antiepileptic, and analgesic properties. Their potential therapeutic role in FM has been investigated in multiple studies, though the quality of evidence remains low.

A systematic review by Kurlyandchik et al. analyzed 10 studies, including three randomized controlled trials (RCTs) and observational studies, encompassing 1,136 patients. The review concluded that medical cannabis (MC) was safe and well tolerated, but its short-term benefits for pain relief were limited. The authors emphasized the need for further studies to determine optimal routes of administration, dosages, and the appropriate THC-to-CBD ratio [42].

Similarly, Strand et al. reviewed four RCTs and five observational studies, involving 564 participants, and found low-quality evidence supporting short-term pain reduction in FM. The authors recommended further research to clarify the efficacy of cannabinoids and the most suitable THC–CBD formulations [43].

More recently, de Carvalho et al. (2024) reviewed 13 studies on the use of medical cannabis in rheumatic diseases, including FM. While most studies reported some degree of benefit, adverse effects were also frequently observed. The authors emphasized the need for additional well-designed clinical trials to assess the impact of cannabis on different symptom domains of FM [44].

Although preliminary findings suggest that medical cannabis may provide some symptom relief in FM, the current evidence remains weak and inconsistent. More high-quality, large-scale studies are necessary to determine their true therapeutic role, optimal dosing strategies, and long-term safety profile in FM management. [45]

Are intravenous therapies effective in the treatment of FM? (especially lidocaine and intravenous ketamine)

Consensus: There is no evidence to recommend the use of intravenous lidocaine and ketamine in the treatment of FM.

Intravenous (IV) lidocaine has been explored as a potential treatment for FM due to its analgesic properties and ability to function as an N-methyl-D-aspartate receptor (NMDAR) antagonist. However, current evidence does not strongly support its use.

A meta-analysis of randomized controlled trials (RCTs) up to 2021, including 62 patients (predominantly women) from four studies, found that IV lidocaine did not significantly improve pain or quality of life compared to placebo [46].

A systematic review from 2022, which analyzed 10 studies involving 461 patients, identified short-term symptom improvements in some cases, though long-term benefits remain uncertain [47]. Given these findings, further well-designed clinical trials are needed to assess the potential role of IV lidocaine in FM management.

Ketamine, an NMDA receptor antagonist, and glutamate receptor modulator, has also been evaluated for its potential analgesic effects in FM.

A review by Pastrak et al. analyzed five placebo-controlled studies and two case reports, examining IV ketamine doses ranging from 0.1 to 0.5 mg/kg. The findings suggested short-term pain reduction, with effects lasting up to seven days in some patients. The authors emphasized the need for larger trials to evaluate long-term efficacy and safety [48].

Similarly, a review by de Carvalho & de Sena (2021), which included six studies, assessed ketamine at doses between 0.1 and 0.5 mg/kg IV or 100 mg/day subcutaneously. While some pain relief was observed, the effects were not sustained over time, and several studies had a risk of bias [49].

Are benzodiazepines and nonbenzodiazepine hypnotics effective in FM?

Consensus: There is no evidence to recommend the use of benzodiazepines or nonbenzodiazepine hypnotics in the treatment of FM.

The use of benzodiazepines in patients with FM is typically aimed at managing comorbid conditions, such as anxiety and sleep disturbances, rather than directly targeting FM pathophysiology or symptoms [50].

Although sedative-hypnotic medications are sometimes mentioned in the context of FM treatment, their therapeutic role is not emphasized in the current literature. They are not considered primary treatment option, as their long-term use may be associated with adverse effects, including tolerance, dependence, and cognitive impairment [51].

Are psychostimulants effective in FM?

Consensus: There is no evidence to recommend the use of psychostimulants in the treatment of FM.

The consensus-seeking system did not identify any relevant studies evaluating the use of psychostimulants in FM.

Psychostimulants, which are primarily indicated for conditions such as attention deficit hyperactivity disorder (ADHD) and narcolepsy, have not been widely studied for FM. The current scientific literature lacks robust evidence to support their efficacy and safety in the management of FM symptoms, and they are not recommended as part of standard treatment protocols. Further high-quality clinical trials would be necessary to determine their potential role in FM management.

## Conclusion

The pharmacological management of fibromyalgia remains a challenge due to its multifactorial pathophysiology and variable treatment responses. This consensus provides updated, evidence-based recommendations to guide rational drug use, ensuring clinical efficacy and safety. While some medications, such as amitriptyline, duloxetine, and pregabalin, demonstrate moderate benefits, many others lack robust evidence for efficacy. A comprehensive, individualized approach integrating pharmacological and non-pharmacological strategies remains essential for optimizing patient outcomes.

## Data Availability

No datasets were generated or analysed during the current study.

## Abbreviations

CBD: Cannabidiol (component of cannabis)
CI: Confidence Interval
CS: Central Sensitization
DLX: Duloxetine
FM: Fibromyalgia
GAB: Gabapentin
HRQoL: Health-Related Quality of Life
IV: Intravenous
LDN: Low-Dose Naltrexone
MC: Medical Cannabis
MLN: Milnacipran
NMDAR: N-Methyl-D-Aspartate Receptor
LDN: Low-Dose Naltrexone
NNH: Number Needed to Harm (measure of adverse effects likelihood)
NNT: Number Needed to Treat (measure of treatment effectiveness)
NSAID: Non-Steroidal Anti-Inflammatory Drug
PGB: Pregabalin
PGIC: Patient Global Impression of Change
PROs: Patient-Reported Outcomes
QoL: Quality of Life
RCT: Randomized Controlled Trial
SBR: Brazilian Society of Rheumatology
SNRI: Serotonin-Norepinephrine Reuptake Inhibitors
SSRI: Selective Serotonin Reuptake Inhibitors
TCA: Tricyclic Antidepressants
THC: Tetrahydrocannabinol (component of cannabis)
TNX-102 SL: Sublingual Cyclobenzaprine Selective Serotonin Reuptake Inhibitors

## References

[1] Siracusa R, Paola RD, Cuzzocrea S, Impellizzeri D. Fibromyalgia: Pathogenesis, mechanisms, diagnosis and treatment options update. Int J Mol Sci. 2021, Apr, 9;22(8):3891. 33918736 10.3390/ijms22083891 PMC8068842

[2] Lahat-Birka N, Boussi-Gross R, Ben Ari A, Efrati S, Ben-David S. Retrospective analysis of fibromyalgia: Exploring the interplay between various triggers and Fibromyalgia’s severity. Clin J PAin. 2024, Oct, 1;40(10):578–87. 39099287 10.1097/AJP.0000000000001236

[3] Heymann RE, et al. Brazilian consensus on the treatment of fibromyalgia. In: Rev bras reumatol. Vol. 50(1): English, Portuguese; 2010 Jan-Feb. p. 56–66. 21125141

[4] Axia.BioFarmacoeconomia e pesquisa em saúde. Available at: http://www.axiabiobrazil.com.

[5] Häuser W, Wolfe F, Tölle T, Uçeyler N, Sommer C. The role of antidepressants in the management of fibromyalgia syndrome: A systematic review and meta-analysis. CNS Drugs. 26(4):297–307. 10.2165/11598970-000000000-00000 22452526

[6] Farag HM, Yunusa I, Goswami H, et al. Comparison of amitriptyline and US food and drug administration-approved treatments for fibromyalgia: A systematic review and network meta-analysis. JAMA Netw Open Open. 5(5):e2212939. 10.1001/jamanetworkopen.2022.12939 PMC9121190 35587348

[7] Häuser W, Bernardy K, Uçeyler N, Sommer C. Treatment of fibromyalgia syndrome with antidepressants: a meta-analysis. JAMA. 2009, Jan, 14;301(2):198–209. 19141768 10.1001/jama.2008.944

[8] Moore RA, Derry S, Aldington D, Cole P, Wiffen PJ. Amitriptyline for neuropathic pain in adults. Cochrane Database Syst Rev. 2012;12), CD008242. 10.1002/14651858.CD008242.pub3 PMC6447238 26146793

[9] Welsch P, Üçeyler N, Klose P, Häuser W. Serotonin and norepinephrine reuptake inhibitors (SNRIs) for fibromyalgia. Cochrane Database Syst Rev. 2018;2), CD010292. 10.1002/14651858.CD010292.pub2 PMC5846183 29489029

[10] . Lederman S, Arnold LM, Vaughn B, Kelley M, Sullivan GM. Efficacy and safety of Sublingual Cyclobenzaprine for the treatment of fibromyalgia: Results from a randomized, double-blind, placebo-controlled trial. Arthritis Care Res (hoboken). 2023;75(11):2359–68. 37165930 10.1002/acr.25142

[11] Tofferi JK, Jackson JL, O’Malley PG. Treatment of fibromyalgia with cyclobenzaprine: a meta-analysis. Arthritis Care Res. 2004;51:9–13. 10.1002/art.20076 14872449

[12] Vaerøy H, Abrahamsen A, Førre Ø, Kåss E. Treatment of fibromyalgia (fibrositis syndrome): a parallel double-blind trial with carisoprodol, paracetamol, and caffeine (somadril comp®) versus placebo. Clin rheumatol. 1989;8(2):245–50. 2667860 10.1007/BF02030081

[13] Moret C, Briley M. Antidepressants in the treatment of fibromyalgia. Neuropsychiatr Dis Treat. 2006;2(4):537–48. 19412502 10.2147/nedt.2006.2.4.537 PMC2671948

[14] Walitt B, Urrútia G, Nishishinya MB, Cantrell SE, Häuser W. Selective serotonin reuptake inhibitors for fibromyalgia syndrome. Cochrane Database Syst Rev. 2015, Jun, 5;2015(6): CD011735. 10.1002/14651858.CD011735 PMC4755337 26046493

[15] F BM, Maffulli N, Eschweiler J, Baroncini A, Bell A, Colarossi G. Duloxetine for fibromyalgia syndrome: a systematic review and meta-analysis. J Orthop Surg Res. 2023, Jul, 17;18(1):504. 37461044 10.1186/s13018-023-03995-z PMC10351165

[16] P CW, N Ü, Klose P, Walitt B, Häuser. Serotonin, and noradrenaline reuptake inhibitors (SNRIs) for fibromyalgia. Cochrane Database Syst Rev. 2018, Feb, 28;2(2): CD010292. 10.1002/14651858.CD010292.pub2 PMC5846183 29489029

[17] Ottman AA, Warner CB, Brown JN. The role of mirtazapine in patients with fibromyalgia: a systematic review. Rheumatol Int. 2018;38(12):2217–24. 29860538 10.1007/s00296-018-4068-3

[18] Samborski W, Lezańska-Szpera M, Rybakowski JK. Effects of antidepressant mirtazapine on fibromyalgia symptoms. Rocz Akad Med BialymSt. 2004;49:265–69. 15631355

[19] Yeephu S, Suthisisang C, Suttiruksa S, Prateepavanich P, Limampai P, Russell IJ. Efficacy and safety of mirtazapine in fibromyalgia syndrome patients: a randomized placebo-controlled pilot study. Ann pharmacother. 2013;47:921–32. 23737510 10.1345/aph.1R725

[20] Miki K, Murakami M, Oka H, Onozawa K, Yoshida S, Osada K. Efficacy of mirtazapine for the treatment of fibromyalgia without concomitant depression: a randomized, double-blind, placebo-controlled phase IIa study in Japan. Pain. 2016;157:2089–96. 27218868 10.1097/j.pain.0000000000000622 PMC4988084

[21] Suttiruksa S, Yeephu S, Prateepavanich P, Suthisisang C. Effects of mirtazapine on quality of life of Thai patients with fibromyalgia syndrome: a double-blind, randomized, placebocontrolled trial. Asian Biomed. 2016;10:435–45.

[22] Welsch P, Bernardy K, Derry S, Moore RA, Häuser W. Mirtazapine for fibromyalgia in adults. Cochrane Database Syst Rev. 2018, Aug, 6;8(8): CD012708. 10.1002/14651858.CD012708.pub2 PMC6513659 30080242

[23] Calandre EP, Morillas-Arques P, Molina-Barea R, Rodriguez-Lopez CM, Rico-Villademoros F. Trazodone plus pregabalin combination in the treatment of fibromyalgia: atwo-phase, 24-week, open-label uncontrolled study. BMC Musculoskelet DisorD. 2011;12:95. 21575194 10.1186/1471-2474-12-95 PMC3112435

[24] Morillas-Arques P, Rodriguez-Lopez CM, Molina-Barea R, Rico-Villademoros F, Calandre EP. Trazodone for the treatment of fibromyalgia: an open-label, 12-week study. BMC Musculoskelet DisorD. 2010;11:204. 20831796 10.1186/1471-2474-11-204 PMC2945951

[25] Sills GJ. The mechanisms of action of gabapentin and pregabalin. Curr Opin Pharmacol. 2006;6(1):108–13. 16376147 10.1016/j.coph.2005.11.003

[26] Lyseng-Williamson KAS, MA. Pregabalin: A review of its use in fibromyalgia drugs. 2008;68:2205–23. 10.2165/00003495-200868150-00009 18840008

[27] Häuser W, Petzke F, Sommer C. Comparative efficacy and harms of duloxetine, milnacipran, and pregabalin in fibromyalgia syndrome. J PAin. 2010;11:505–21. 20418173 10.1016/j.jpain.2010.01.002

[28] Derry S, Cording M, Wiffen PJ, Law S, Phillips T, Moore RA. Pregabalin for pain in fibromyalgia in adults. Cochrane Database Syst Rev. 2016, Sep, 29;9(9): CD011790. 10.1002/14651858.CD011790.pub2 PMC6457745 27684492

[29] Cooper TE, Derry S, Wiffen PJ, Moore RA. Gabapentin for fibromyalgia pain in adults. Cochrane pain, palliative and supportive care group, ed. Cochrane Database Syst Rev. 2017, Jan, 3;1(1): CD012188. 10.1002/14651858.CD012188.pub2 PMC6465053 28045473

[30] Bruun-Plesner K, Blichfeldt-Eckhardt MR, Vaegter HB, et al. Low-dose naltrexone for the treatment of fibromyalgia: Investigation of dose‒response relationships. Pain Med. 2020;21(10):2253–61. 32068870 10.1093/pm/pnaa001

[31] Partridge S, Quadt L, Bolton M, et al. A systematic literature review on the clinical efficacy of low dose naltrexone and its effect on putative pathophysiological mechanisms among patients diagnosed with fibromyalgia. Heliyon. 2023;9(5):e15638. 37206027 10.1016/j.heliyon.2023.e15638 PMC10189400

[32] Yang J, Shin KM, Do A, Bierle DM, Abu Dabrh AM, Yin Z, Bauer BA, Mohabbat AB. The safety and efficacy of low-dose naltrexone in patients with fibromyalgia: A systematic review. J Pain Res. 2023;16:1017–23. 36974308 10.2147/JPR.S395457 PMC10039621

[33] da Rocha Ap, Mizzaci CC, Nunes Pinto ACP, da Silva Vieira Ag, Civile V, Trevisani VFM. Tramadol for management of fibromyalgia pain and symptoms: Systematic review. Int J Clin Pract. 2020, Mar;74(3):e13455. 31799728 10.1111/ijcp.13455

[34] Roskell NS, Beard SM, Zhao Y, et al. A meta-analysis of pain response in the treatment of fibromyalgia. Pain Pract. 2011;11:516–27. 21199320 10.1111/j.1533-2500.2010.00441.x

[35] Goldenberg DL, Clauw DJ, Palmer RE, Clair AG. Opioid use in fibromyalgia: a cautionary tale. Mayo Clin Proc. 2016;91(5):640–48. 26975749 10.1016/j.mayocp.2016.02.002

[36] Peng X, Robinson RL, Mease P, et al. Long-term evaluation of opioid treatment in fibromyalgia. Clin J PAin. 2015;31(1):7–13. 24480913 10.1097/AJP.0000000000000079

[37] Fitzcharles MA, Ste-Marie PA, Gamsa A, Ware MA, Shir Y. Opioid use, misuse, and abuse in patients labeled as fibromyalgia. Am J Med. 2011;124(10):955–60. 21962316 10.1016/j.amjmed.2011.05.031

[38] Bao JD, Rosser MA, Park SH, Baker AK, Martucci KT. Interplay between noxious heat sensitivity and temporal summation magnitude in patients with fibromyalgia and long-term opioid use. Front neurosci. 2023;17:1275921. 37901425 10.3389/fnins.2023.1275921 PMC10600517

[39] Choy E, Marshall D, Gabriel ZL, et al. A systematic review and mixed treatment comparison of the efficacy of pharmacological treatments for fibromyalgia. Semin Arthritis Rheum. 2011;41:335–45. 21868065 10.1016/j.semarthrit.2011.06.003

[40] Derry S, Wiffen PJ, Häuser W, et al. Oral nonsteroidal anti-inflammatory drugs for fibromyalgia in adults. Cochrane pain, palliative and supportive care group, ed. Cochrane Database Syst Rev. 2017, 2020, 27;3(3): CD012332. 10.1002/14651858.CD012332.pub2 PMC6464559 28349517

[41] Thorpe J, Shum B, Moore RA, Wiffen PJ, Gilron I. Combination pharmacotherapy for the treatment of fibromyalgia in adults. Cochrane Database Syst Rev. 2018, Feb, 19;2(2): CD010585. 10.1002/14651858.CD010585.pub2 PMC6491103 29457627

[42] Kurlyandchik I, Tiralongo E, Schloss J. Safety and efficacy of medicinal cannabis in the treatment of fibromyalgia: A systematic review. J Altern Complement Med. 2021, Mar;27(3):198–213. 33337931 10.1089/acm.2020.0331

[43] Strand NH, Maloney J, Kraus M, Wie C, Turkiewicz M, Gomez DA, Adeleye O, Harbell MW. Cannabis for the treatment of Fibromyalgia: A systematic review. Biomedicines. 2023, Jun, 2;11(6):1621. 37371716 10.3390/biomedicines11061621 PMC10295750

[44] de Carvalho Jf, Ribeiro MFLDS, Skare T. Cannabis therapy in rheumatological diseases: A systematic review. N Clin Istanb. 2024, Jul, 22;11(4):361–66. 10.14744/nci.2023.43669 PMC11331211 39165706

[45] Khurshid H, Qureshi IA, Jahan N, Went TR, Sultan W, Sapkota A, Alfonso M. A systematic review of fibromyalgia and recent advancements in treatment: Is medicinal cannabis a new hope? Cureus. 2021, Aug, 20;13(8):e17332. 34567876 10.7759/cureus.17332 PMC8451533

[46] Nm L, Hilal FM, Bashawyah A, et al. Efficacy of acupuncture, intravenous lidocaine, and diet in the management of patients with fibromyalgia: A systematic review and network meta-analysis. Healthcare. 2022;10(7):1176. 35885703 10.3390/healthcare10071176 PMC9320380

[47] de Carvalho Jf, Skare TL. Lidocaine in fibromyalgia: A systematic review. World J Psychiatry. 2022;12(4):615–22. 35582338 10.5498/wjp.v12.i4.615 PMC9048454

[48] Pastrak M, Abd-Elsayed A, Ma F, Vrooman B, Visnjevac O. Systematic review of the use of intravenous ketamine for fibromyalgia. Ochsner J. 2021;21(4):387–94. 34984054 10.31486/toj.21.0038 PMC8675611

[49] Carvalho Jf B, Sena Ep B. Ketamine in fibromyalgia: a systematic review. Adv rheumatol. 2024;64(1):54. 39075628 10.1186/s42358-024-00393-9 PMC13488823

[50] Kim SC, Landon JE, Solomon DH. Clinical characteristics and medication uses among fibromyalgia patients newly prescribed amitriptyline, duloxetine, gabapentin, or pregabalin. Arthritis Care Res (hoboken). 2013;65(11):1813–19. 23861291 10.1002/acr.22071 PMC4059353

[51] Di Franco M, Iannuccelli C, Atzeni F, Cazzola M, Salaffi F, Valesini G, Sarzi Puttini P. Pharmacological treatment of fibromyalgia. Clin Exp rheumatol. 2010, Nov-Dec;28(6 Suppl 63):S110–6. 21176430

